# Adiponectin, resistin and IL-6 plasma levels in subjects with diabetic foot and possible correlations with clinical variables and cardiovascular co-morbidity

**DOI:** 10.1186/1475-2840-9-50

**Published:** 2010-09-13

**Authors:** Antonino Tuttolomondo, Sergio La Placa, Domenico Di Raimondo, Chiara Bellia, Antonietta Caruso, Bruna Lo Sasso, Giovanni Guercio, Giuseppe Diana, Marcello Ciaccio, Giuseppe Licata, Antonio Pinto

**Affiliations:** 1 Dipartimento Biomedico di Medicina Interna e Specialistica Università degli Studi di Palermo, Italy; 2 Dipartimento di Biopatologia e Biotecnologie Mediche e Forensi, Università degli studi di Palermo, Italy; 3 Dipartimento di Chirurgia Generale, d'Urgenza e dei Trapianti d'Organo-Università degli Studi di Palermo, Italy

## Abstract

**Introduction:**

It is very suggestive that diabetic foot is characterized by a pronounced inflammatory reaction and the pathogenic significance of this inflammation has received little attention. On this basis the aim of our study was to evaluate plasma levels of adiponectin, resistin and IL-6 in subjects with diabetic foot in comparison with subjects without foot complications.

**Materials and methods:**

We recruited 34 subjects with type 2 diabetes mellitus and foot ulceration hospitalized for every condition related to diabetic disease, but not for new vascular events *(group A). *As controls we recruited 37 patients with type 2 diabetes mellitus without foot ulceration *(group B) *hospitalized for every condition related to diabetic disease, but not for new vascular events. Adiponectin, Resistin and IL-6 serum levels were evaluated.

**Results:**

Subjects of group A showed lower median plasma levels of adiponectin [7.7450 (4.47-12.17) μg/ml vs 8.480 (5.15-12.87) μg/ml], higher median plasma levels of IL-6 [3.21 (1.23-5.34) pg/ml vs 2.73 (1.24-3.97 pg/ml)] and of resistin [3.860 (2.96-6.29 ng/ml) vs 3.690 (2.,37-6.5 ng/ml)].

**Conclusion:**

Our study demonstrated that diabetic subjects with diabetic foot showed in comparison with diabetics without diabetic foot higher IL-6 and resistin plasma levels, lower adiponectin plasma levels.

## Introduction

In diabetes exists a complex interrelationship of various inflammatory variables with metabolic disorders and their effect on cardiovascular system.

Simplified explanation can be that inflammation increases insulin resistance, which in turn leads to obesity while perpetuating diabetes, high blood pressure, prothrombotic state and dyslipidaemia [1]. Some studies [2-4] have produced data suggesting an interplay between hormones, cytokines and resistin.

Nevertheless, adiponectin, the most abundant adipocytokine, was found to be decreased in conditions such as obesity, type 2 diabetes, and coronary heart disease (CHD) [5-7]. In this context, hypoadiponectinemia was associated with low HDL-cholesterol (HDL-C) concentrations [8], decreased LDL particle size [6], and increased markers of systemic inflammation [9].

Jeffcoate et al [10] al, suggested that an inflammatory cascade through increased expression of proinflammatory cytokines, including TNF-α and interleukin-1β exist in diabetic foot, whereas no study, to our knowledge, evaluated the role of adiponectin, resistin and immune-inflammatory biomarkers such as inflammatory cytokines in patients with diabetic foot in comparison with diabetic subjects without foot complications.

On this basis the aim of our study was to evaluate plasma levels of adiponectin, resistin and IL-6 in subjects with diabetic foot in comparison with subjects without foot complications.

## Materials and methods

We recruited 34 subjects with type 2 diabetes mellitus and foot ulceration (group A) hospitalized between 2006 and 2008 every for every condition related to diabetic disease (*decompensated diabetes, hypoglycemia, clinical revaluation for foot ulceration)*, but not for new vascular events (group A) at the Internal and Specialist Medicine Department and at Diabetic Foot Surgery Service of the Policlinico P. Giaccone Hospital of Palermo. We also recruited 37 patients with type 2 diabetes mellitus without foot ulceration (group B) admitted to our department for other causes between 2006 and 2008. The study was carried out in accordance with the principles of the Declaration of Helsinki as revised in 2001. All patients gave informed consent to take part in this research

Foot ulcer was defined as a full-thickness skin defect that required ≥14 days for healing [11].

Every subject with diabetic foot was matched for age (± 3 years), sex, and cardiovascular risk factor prevalence with one control subject. Patients with inflammatory or infectious diseases, autoimmune and rheumatic diseases, cancer, haematological diseasesand severe renal or liver failure, as well as those who were under treatment with anti-inflammatory drugs, were excluded. We also excluded patients with fever and recent venous thromboembolism

A physical examination with emphasis on the lower limbs was performed by research operators, who assessed the presence of the following characteristics: hammer/claw toe, Charcot deformity, hallux limitus, prominent metatarsal heads, hallux valgus, bony prominences, and ankle and halluxobility measured with a goniometry.

Type 2 diabetes mellitus was determined using a clinically based algorithm that considered age at onset, presenting weight and symptoms, family history, onset of insulin treatment, and history of ketoacidosis.

Hypertension was defined according to the 1993 World Health Organization criteria (systolic blood pressure ≥140 mm Hg and/or diastolic blood pressure ≥90 mm Hg in subjects who are not taking antihypertensive medication or antihypertensive treatment yet present on admission) [12]. Hypercholesterolemia was defined as total serum cholesterol ≥200 mg/dL and hypertriglyceridemia as total serum triglyceride ≥150 mg/dL on the basis of the National Cholesterol Education Program-Adult Treatment Panel III reports [13,14] that define this cutoff for optimal total serum cholesterol and triglyceride levels. All patients had blood pressure, serum glucose, creatinine, serum uric acid, serum cholesterol levels, serum triglyceride levels, and urinary albumin excretion (UAE) values measured on admission to the hospital.

The ankle-brachial index (ABI) was calculated as the ratio of the ankle systolic pressure (defined as the higher of the dorsalis pedis or posterior tibialis measurements) divided by the higher brachial systolic pressure. Subjects were classified as having PAD when they had an ABI ≤0.9 and/or when they had undergone a peripheral arterial bypass or amputation [15].

Coronary artery disease was determined on the basis of a history of physician-diagnosed angina, myocardial infarction, or any previous revascularization procedure assessed by a questionnaire. Cerebrovascular disease (TIA/ischemic stroke) was assessed by history, specific neurologic examination executed by specialists, and hospital or radiological (brain computed tomography or brain magnetic resonance) records of definite TIA or stroke.

Neuropathy was detected by physical examination, and medical history reviews of the patient.

### Blood collection and laboratory analysis

Blood samples were obtained in the non-fasting state. After 10 min of rest in the supine position, vital signs were recorded and blood samples were collected from the antecubital vein. EDTA-anticoagulated peripheral blood was drawn from each patient within 12 h from symptom onset. Serum and plasma were immediately separated by centrifugation and stored in aliquots at -80°C until analysis.

Adiponectin, Resistin and IL-6 serum levels were measured by enzyme-linked immunosorbent assay (ELISA) method according to manufacturer suggestions. Regarding to Adiponectin and Resistin determination, high sensitivity kits (Biovendor) were used; IL-6 was determined by the Diaclone ELISA kit.

Regarding the sensitivity of Adiponectin test (Biovendor), the analitical limit of detection was 0.6 microg/ml; intra- and interassay coefficients of variation (%) were 4.1 and 4.0, respectively.

For Resistin assay (Biovendor), the analitical limit of detection was 0.1 ng/ml; intra- and interassay CV (%) were 2.8 and 5.1, respectively.

For IL-6 assay (Diaclone) the analitical limit of detection was 0.2 pg/ml; intra- and interassay CV (%) were 4.2 and 7.7, respectively.

### Statistical Analysis

Results are expressed as median (lower Quartile ↔ upper Quartile) for continuous variables and percentages for categorical data, with *P *< 0.05 considered significant. Analysis of normality was performed with the Shapiro-Wilk W test. Non-normally distributed data were logarithmically (Log10) transformed before analysis. The relationship between IL-6, adiponectin and resistin and other clinical and laboratory variables was analyzed using nonparametric methods (Spearman p correlations) after correction for age and gender.

Hazard ratios for the presence of foot ulcer were determined by univariate and multivariate Cox proportional hazards regression analyses with data presented as hazard ratio with 95% confidence interval. Initial univariate analyses identified demographic, clinical and laboratory variables that independently predicted foot ulcer presence.

According to sample size calculation a sample size of 30 patient-control pairs had 80% power at the 5% significance level to detect a 10% difference in selected biomarker plasma levels and arterial stiffness indexes between control subjects and patients and between each subtype of stroke.

## Results

Baseline characteristic of subjects with diabetic foot in comparison with subjects without diabetic foot are given in table 1.

**Table 1:** General and demographic variables in cases and controls

	pts with diabetic foot	pts without diabetic foot	p
**n**:	34	37	0.75

**Age**	66.7 ± 8.5	66.9 ± 7.9	0.027

**Sex Male (n/%)**	16 (47,1%)	15 (41,7%)	0.41

**Diabetes duration**			
**<10 yrs**	7 (20,6%)	21 (58,3%)	0.027
**=10 yrs**	8 (23.5%)	11 (30.6%)	0.045
**=20 yrs**	19 (55.9)	4 (11.1%)	<0.001

**treatment Diet (n/%)**	4 (11,8)	3 (8.3%)	0.65
**Antidiabetici orali (n/%)**	3 (8.8)	10 (27.8%)	<0.001
**Mixed (n/%)**	6 (17.5)	13 (36,1%)	<0.001
**Insulin (n/%)**	21 (61.8)	10 (27.8)	<0.001

**Smoking (n/%)**	7 (20,6)	9 (25)	0.71

**Hypertension (n/%)**	20 (58,8)	25 (69.4)	0.041

**Dyslipidaemia (n/%)**	14 (41,2)	16 (44.4)	0.35

**Obesity (n/%)**	19 (55,9)	13 (36.1)	0.021

**Chronic renal failure (n/%)**	15 (44,1)	13 (36.1)	0.064

**Mycroalbuminuria (n/%)**	22 (64,7)	6 (14,7)	<0.001

**Retinopathty (n/%)**	19 (55,9)	36 (100)	<0.001

**PAD (n/%)**	10 (29.41)	9 (25)	0.54

**CAD (n/%)**	17 (50)	7 (19,4)	<0.001

**TIA/Stroke (n/%)**	14 (41.17)	6 (16.66)	0.021

**Other district atherosclerosis (n/%)**	28 (82.35	21 (58.33)	<0.001

**Artropathy (n/%)**	11 (32,4%)	2 (5.6%)	<0.001

**Neuropathy (n/%)**	25 (73.52)	14 (38.88%)	<0.001

**Diabetic foot grade**			
**Grade 0**	1 (2,9%)	-	
**Grade 1**	**6 (17,6%)**	-	
**Grade 2**	**8 (23,5%)**	-	
**Grade 3**	**10 (29,4%)**	-	
**Grade4**	**4 (11,8%)**	-	
**Grade 5**	**1 (2,9%)**	-	
**Grade 6**	**4 (11,8%)**	-	

Data are expressed as median and interquartile (lower and upper quartile)

PAD: peripheral artery disease; CAD: coronary artery disease

In group A 47.1% of subjects was male, while 41.7% of subjects in group B were male. In group A 97.1% of subjects had diabetes mellitus type 2, while in group B type 2 diabetes was present in 97.2% of subjects.

Regarding the duration of diabetes of 20.6% of subjects in group A vs 58.3% of subjects in group B could be diabetic by <10 years, the 23.5% vs 30.6% for about 10 years, the 55.9% in group A vs 11.1% in group B by >10 years.

The 11.8% of subjects in group A vs 8.3% of subjects in group B was treated with diet, 8.8% vs 27.8% was in treatment with oral anti-diabetic, 61.8% vs 27.8% was in treatment with insulin. With regard of cardiovascular risk factors, 20.6% of subjects in group A vs 25% of subjects in group B was a smoker, 58.8% vs 69.4% had hypertension, 41.2% vs 44.4% had dyslipidaemia, 59.4% vs 36.1% had obesity and 64.7% vs 14.7% showed microalbuminuria.

Regarding the prevalence of previous vascular morbidity 29.4% of subjects in group A vs 25% of subjects in group B had PAD, 50% vs 19.4% had ischemic heart disease, 41.17% vs 16.66% had a previous TIA or stroke, 44.1% of subjects in group A vs 36.1% of subjects in group B presented a chronic renal failure, while 73.52% vs 38.8% had a neuropathy Subjects in group A also presented, in comparison with those in group B, increased median levels of HbA1c [8 (7.28-9.40)% vs, 8.5 (6.10-8.00)% ], CRP [4 (2,25-5.15) mg/dL vs 2, 25 (1.90-3.08) mg/dL)], total cholesterol [215.50 (166.50-243,00) mg/dL vs 204.00 (185.50 to 210.00 mg/dL)], LDL [121.70 (98.75-148.75) mg/dL vs 104.50 (78.00-123.00 mg/dL], white blood cells [12675 (10775.00-14140.00 mg/dL vs 10700 (8850-12027.50) mg/dL ] (see table 2).

**Table 2:** Laboratory variables in cases and controls

	Diabetic foot patients	Diabetics without foot complications	p
**HbA1c**	8 (7.28-9.40)	6.85 (6.10-8.00)	**0.018**

**CRP**	4 (2.25-5.15)	2.25 (1.90-3.08)	**0.041**

**Total cholesterol (mg/dl)**	215.50 (166,50-243,00)	204.00 (185,50-210,00)	0.054

**LDL cholesterol (mg/dl)**	121.70 (98,75-148,75)	104.50 (78,00-123,00)	**0.032**

**Tryglicerids (mg/dl)**	160.50 (119,50-209,25)	180.50 (144,50-199,00)	**0.012**

**Globuli bianchi**	12.675 (10775,00-14140,00)	10.700 (8850,00-12027,50)	**0.032**

**Adiponectin (μg/ml)**	7.1450 (4.47-12.17)	8.480 (5.15-12.87)	**0.022**

**Resistin (ng/ml)**	5.160 (2.96-6.29)	3.290 (2.37-6.5)	**0.021**

**IL-6 (pg/ml)**	3.21 (1.23-5.34)	2.13 (1.24-3.97)	**0.033**

Demographic and anamnestic data are expressed as n° (percentage); HbA1c: Hemoglobin A1c; CRP: C-reactive protein; IL-6: Interleukin-6

Finally, subjects of group A showed lower median plasma levels of adiponectin [7.7450 (4.47-12.17) μg/ml vs 8.480 (5.15-12.87) μg/ml], higher median plasma levels of IL-6 [3.21 (1.23-5.34) pg/ml vs 2.73 (1.24-3.97 pg/ml)] (see table 2) and higher median plasma levels of resistin [5.160 (2.96-6.29 ng/ml) vs 3.690 (2.,37-6.5 ng/ml)](see table 2).

### Correlation analysis

There was a significant positive correlation, corrected for age, and gender, between IL-6 and diabetes duration (r = 0.29; p = 0.027), hypertension (r = 0.28; p = 0.030), dyslipidaemia (r = 0.25; p = 0.037), mycroalbuminuria (r = 0.29; p < 0.05), retinopathy (r = 0.26; p = 0.021), PAD (r = 0.30; p < 0.05) previous TIA/Stroke (r = 0.32; p < 0.0001), neuropathy (r = 0.34; p < 0.0001, diabetic foot grade, (r = 0.30; p < 0.0001)(see table 3).

There was a significant positive correlation corrected for age and gender, between resistin and diabetes duration (r = 0.35;p < 0.05) hypertension (r = 0.22;p = 0.022), dyslipidaemia (r = 0.027; p = 0.041), BMI (r = 0.29; p = 0.022), mycroalbuminuria (r = 0.30; p < 0.05), retinopathy (r = 0.26; p = 0.031), previous TIA/stroke (r = 0.31; p < 0.0001), neuropathy (r = 0.33; p < 0.0001, diabetic foot grade (r = 0.32 = < 0.0001)(see table 3).

There was a significant negative correlation between adiponectin and diabetes duration (r = -0.37; p < 0.05); hypertension (r = -0.32; p < 0.05), dyslipidaemia (r = 0.39; p < 0.0001), retinopathy r = -0.28; p = 0.40); PAD (r = 0.20; p = 0.021), previous TIA/Stroke (r = -0.30; p = 0.021), neuropathy (r = -0.31; p > 0.0001), diabetic foot grade (r = -0.29; p < 0.05)(see table 3).

**Table 3:** Correlation analysis between adiponectin, resistin and IL-6 plasma levels and laboratory and clinical variables in patients with diabetic foot

	Adiponectin		Resistin		IL-6	
**Variable**	**r**	**p**	**r**	**p**	**r**	**p**

**Sex (male)**	0.11	0.87	0.10	0.83	0.14	0.79

**Diabetes duration**	**-0.37**	**<0.05**	**0.33**	**<0.05**	**0.29**	**0.027**

**Smoking**	0.12	0.67	0.09	0.12	0.09	0.12

**Hypertension**	**-0.32**	**<0.05**	**0.29**	**0.022**	**0.28**	**0.020**

**Dyslipidaemia**	**-0.29**	**0.032**	**0.27**	**0.041**	**0.25**	**0.037**

**BMI**	**-0.39**	**<0.0001**	**0. 29**	**0.022**	0.10	0.20

**Chronic renal failure**	0.11	0.23	0.17	0.81	0.19	0.88

**Mycroalbuminuria**	0.11	0.08	0.13	0.64	**0.29**	**<0.05**

**Retinopathy**	**-0.28**	**0.040**	**0.26**	**0.031**	**0.26**	**0.021**

**PAD**	0.10	0.55	0.11	0.34	**0.30**	**<0.05**

**CAD**	0.14	0.77	0.11	0.67	0.11	0.67

**Previous TIA/Stroke**	**-0.29**	**<0.05**	**0.31**	**<0.0001**	**0.32**	**<0.0001**

**Neuropathy**	**-0.31**	**<0.0001**	**0.33**	**<0.0001**	**0.34**	**<0.0001**

**Diabetic foot grade**	**-0.29**	**<0.05**	**0.32**	**<0.0001**	**0.30**	**<0.0001**

BMI: body mass index; PAD: peripheral artery disease; CAD: coronary artery disease

### Univariate analysis

On univariate analysis, age, diabetes duration, hypercholesterolemia, hypertension, mycroalbuminuria, retinopathty, PAD, CAD, previous TIA/Stroke, Il-6 plasma levels, resistin plasma levels were significantly associated with diabetic foot presence, whereas adiponectin plasma levels were negatively associated with diabetic foot (see table 4)

**Table 4:** Cox regression analysis of clinical and laboratory variables associated with diabetic foot presence

	Univariate analysis			Multivariate analysis		
	**Regression** **coefficient**	**Hazard ratio** **(95% CI)**	**P value**	**Regression** **coefficient**	**Hazard ratio** **(95% CI)**	**P value**

**Age**	**0.69**	**2.32 (1.78-5.19)**	**0.021**	**0.49**	**2.12 (182-4.48)**	**0.031**

Diabetes duration (y)	**1.56**	**7.26 (3.80-13.87)**	**<0.001**	**1.21**	**5.67 (2.70-8.34)**	**<0.001**

Hypercholesterolemia	**0.59**	**1.55 (1.16-2.64)**	**0.21**	**0.44**	0.87 (0.166-1.62)	0.42

**Hypertension (n/%)**	**0.32**	0.78 (1.70-4.34)	0.33	0.39	0.67 (0.30-1.12)	0.71

**Obesity (n/%)**	**0.32**	0.86 (0.31-1.23)	0.23	0.29	0.77 (0.42-0.98)	0.56

**Chronic renal failure (n/%)**	**0.11**	0.89 (0.53-1.31)	0.35	0.11	0.59 (0.21-0.89)	0.71

**Mycroalbuminuria (n/%)**	**0.41**	**1.87 (1.32-3.51)**	**0.018**	**0.41**	**1.55 (1.11-2.99)**	**0.032**

**Retinopathty (n/%)**	**0.33**	**2.31 (1.78-4.78)**	**0.012**	**0.33**	**1.99 (1.45-3.24)**	**0.021**

**PAD (n/%)**	**0.61**	**3.5 (2.7-4.1)**	**<0.005**	**0.59**	**2.9 (2.1-3.8)**	**<0.005**

**CAD (n/%)**	**0.54**	**1.6 (1.4--2.7)**	**0.033**	0.54	0.89 (0.5-1.1)	0.56

**TIA/Stroke (n/%)**	**0.42**	**1.4 (1.21-2.09)**	**0.041**	0.42	0.78 (0.33-0.91)	0.44

**IL-6**	**1.12**	**4.8 (3.1-5.5)**	**<0.005**	**0.98**	**3.6 (2.8-4.1)**	**<0.005**

**Resistin**	**0.49**	**2.1 (1.4-3.9)**	**0.041**	**0.43**	**1.78 (1.4-2.9)**	**0.031**

**Adyponectin**	**-0.29**	**-3.4 (-1.9-3.6)**	**<0.001**	**-0.29**	**-2.9 (-1.5-3.4)**	**<0.001**

### Multivariate analysis

At multivariate analysis **only **age, diabetes duration, hypercholesterolemia, hypertension, mycroalbuminuria, retinopathty, PAD, IL-6 plasma levels, resistin plasma levels were significantly associated with diabetic foot presence, whereas adiponectin plasma levels were negatively associated with diabetic foot (see table 4)

## Conclusion

Our study demonstrated that diabetic subjects with diabetic foot showed in comparison with diabetics without diabetic foot higher IL-6 and resistin plasma levels and lower adiponectin plasma levels.

Resistin, although postulated to contribute to insulin resistance, may also contribute to inflammatory responses. Early investigations into the role of resistin as an inflammatory factor demonstrated that LPS (lipopolysaccharide) up-regulated resistin expression in rat WAT, 3T3-L1 adipocytes and human monocytes [16]. Although initial rodent studies have produced discrepancies as to whether pro-inflammatory cytokines may regulate resistin, several recent human studies have supported the concept of inflammatory cytokine mediation of resistin [17,18].

Moreover, Osawa et al [19] reported that elevated serum resistin concentration appears to be an independent risk factor for ischemic stroke, especially lacunar and atherothrombotic infarction in the general Japanese population. In particular in this study authors showed that the combination of high resistin and the presence of either diabetes or hypertension increased the risk of ischemic stroke.

Our findings concerning the higher plasma levels IL-6 plasma levels and resistin in diabetic subjects with foot ulceration in comparison with diabetics without foot complications may confirm this topics. A recent study by Reilly and co-workers [20] suggested resistin as a metabolic link between inflammation and atherosclerosis.

In contrast with resistin, adiponectin, known to enhance insulin sensitivity and reduce atherosclerotic plaques, suppressed a resistin-mediated rise in VCAM-1 and ICAM-1 [21].

Adiponectin levels can be assessed by either of three variables: total adiponectin, HMWA and the SA index. Recentlly Almeda-Valdes et al. [22] showed that total adiponectin, HMWA and the SA index had similar utility for the identification of the metabolic abnormalities. This finding may stimulate the use of adiponectin in clinical and epidemiological settings because the measurement of total adiponectin is better standardized, cheaper and more accessible than the other two approaches.

Hypoadiponectinaemia can be viewed as an early sign of a complex cardiovascular risk factor predisposing to the atherosclerosis process as well as a contributing factor accelerating the progress of the atherosclerotic plaque. Adiponectin exhibits anti-inflammatory and atheroprotective actions in various tissues by suppressing the expression of vascular adhesion molecules and scavenger receptors, reducing the expression of the inflammatory cytokine TNF-a, raising NO production and suppressing the proliferation and migration of smooth muscle cells [23].

To this date, two receptors have been identified that mediate adiponectin's actions in fatty-acid oxidation and glucose uptake, namely ADIPOR1 and ADIPOR2 [24].

Very recently Halvatsiotis et al [25] have demonstrated for the first time that a sequence variant in the intron 5 of the ADIPOR2, rs767870 among the eight studied, is associated with cardiovascular disease in a population of Greek individuals.

Our findings of lower median plasma levels of adiponectin in subjects with diabetic foot could confirm this issue. Furthermore we observed a significant negative correlation between adiponectin plasma levels and some cardiovascular risk factors such as hypertension, dyslipidaemia and clinical variables indicating previous cardiovascular morbidity such as previous TIA/Stroke and incident vascular morbidity such as neuropathy, microalbuminuria and PAD and these findings further suggest a possible role of hypo-adiponectinaemia as a putative marker of cardiovascular morbidity both prevalent and incident.

As several cytokines are also produced by adipose tissue [26] it was postulated that an "adipo-vascular" axis [27] may contribute to the increased risk of cardiovascular events in patients with type 2 diabetes. In patients with diabetic foot this "adipo-vascular axis"expression in lower plasma levels of adiponectin and higher plasma levels of IL-6 could be linked to foot ulcers pathogenesis by microvascular and inflammatory mechanisms.

Indeed recent studies suggest that adiponectin may play a role in the modulation of inflammatory vascular response by inhibiting the expression of adhesion molecules on endothelial cells [28], inhibiting endothelial cell NF-kB signaling [29], and suppressing macrophage function [30,31].

Other studies showed that adiponectin suppressed the TNF-*α*-stimulated expression of E-selectin, VCAM-1 and ICAM-1 in human endothelial cells [28,29]. This suggests further that adiponectin may be vasoprotective and negatively modulate the atherogenic processes.

Recently Zietz et al [30] reported that low levels of adiponectin are associated with low levels of HDL-cholesterol and might represent an independent cardiovascular risk factor, whereas high levels of adiponectin are associated with high levels of HDL-cholesterol indicating a protective risk profile.

Furthermore we observed significant either positive (for IL-6 and resistin) and negative (for adiponectin) correlations in subjects with diabetic foot between these immuno-inflammatory and metabolic markers and some clinical and laboratory variables and these correlation furtherly underline the relationships with inflammatory background.

Recently our group underlined [31] the role of diabetic foot syndrome (DFS) to predict cardiovascular morbidity in diabetic patients, even after correction for other well-known cardiovascular risk factors. In our study both univariate and multivariate analysis showed the predictive positive role of resistin and IL-6 plasma levels and a negative one of adyponectin towards diabetic foot presence.

These findings furtherly underline the importance of inflammatory and metabolic "milieu" such as cytokines and adipose hormones in foot complications in diabetics as already reported for other vascular complications of diabetes [11,32,33].

A possible limitation our study is that we evaluated only IL-6 as a inflammatory marker. Previous studies have shown the relationship between inflammatory cytokines and cardiovascular morbidity in diabetic patients. Tuttle et al [34] showed that both IL-6 and TNF-α are chronically increased in diabetic women with and without CVD compared to nondiabetic women. The additive concentration of cytokines in diabetes and CVD suggests a common inflammatory state in both diabetes and CVD. Makino et al [32]. reported that serum levels of TNF-α and vascular endothelial growth factor (VEGF) were elevated in diabetic patients with microangiopathy and endothelial dysfunctio. Nevertheless owing to the fact that the mechanisms that control expression of different cytokines are often related and TNF-α stimulates expression of IL-1 and IL-6, and IL-1 can induce both IL-6 and TNF-α, to evaluate only IL-6 expression in our patients with diabetic foot could be a sufficient proof of immuno-inflammatory activation in diabetic patients with foot complications.

## Competing interests

The authors declare that they have no competing interests.

## Authors' contributions

***Study design ***AT, GL, AP. ***Acquisition of data: ***AT, S L, GG, G D***. Analysis and interpretation of data: ***AT, DD, SL, CB, BL, AC, GL, AP, MC. ***Manuscript preparation: ***A.T, SL, DD, GL, AP. All authors read and approved the final manuscript'
